# Editorial: Livestock and its role in the emergence, spread, and evolution of antimicrobial resistance: animal-to-human or animal-to-environment transmission

**DOI:** 10.3389/fvets.2023.1270955

**Published:** 2023-08-29

**Authors:** Eliana Guedes Stehling, William Calero-Cáceres, Kohei Makita, João Pedro Rueda Furlan

**Affiliations:** ^1^Department of Clinical Analyses, Toxicology and Food Science, School of Pharmaceutical Sciences of Ribeirão Preto, University of São Paulo, Ribeirão Preto, Brazil; ^2^UTA-RAM-One Health, Department of Food and Biotechnology Science and Engineering, Universidad Técnica de Ambato, Ambato, Ecuador; ^3^Veterinary Epidemiology Unit, Graduate School of Veterinary Medicine, Rakuno Gakuen University, Ebetsu, Japan

**Keywords:** bacterial pathogens, antimicrobial resistance, molecular epidemiology, genomic evolution, food-producing animals, One Health

The emergence of multidrug-resistant bacterial pathogens is a global multifactorial and multisectoral problem. The intensive use of antimicrobials in livestock has been associated with an increase in antimicrobial resistance (AMR). In this regard, antimicrobial-resistant strains are constantly emerging from this sector, raising concerns about animal-to-human and animal-to-environment transmission. This Research Topic includes original, brief report, and review research focused on AMR in the veterinary sector.

## Cross-species and inter-host transmission of multidrug-resistant gram-negative bacteria

Ramírez-Castillo et al. discussed the spread of carbapenem resistance in terrestrial food-producing animals, seafood, aquaculture, wildlife, companion animals, and their environments. Overall, poultry, swine, and cattle have carried acquired carbapenemases, including KPC, NDM, IMP, VIM, and OXA. These carbapenemases were occurred mainly in *Enterobacterales, Acinetobacter* species, and *Pseudomonas aeruginosa*. In seafood and aquaculture, intrinsic or acquired carbapenemases have been detected in *Enterobacterales, Shewanella algae, Vibrio* species, and clinically important non-fermenting gram-negative bacilli. A high prevalence of clinically significant carbapenemases has been observed in wildlife and companion animals. Furthermore, carbapenemases were also identified in their surrounding environments. These findings show the spread of carbapenem-resistant strains in the veterinary sector and highlight the cross-species transmission of carbapenem resistance.

In Andaman and Nicobar Islands of India, the occurrence of β-lactamase-producing *Enterobacteriaceae* strains from broilers and native fowl was investigated by Bhowmick et al.. Among the identified species, *Klebsiella pneumoniae* was the most prevalent, followed by *Salmonella enterica*, and *Escherichia coli*. These species showed resistance to several antimicrobials, highlighting the β-lactams agents. In this regard, β-lactamase-encoding genes, including *bla*_CTX − M_, were identified. The rate of β-lactamase-producing strains was significantly higher in Andaman than in Nicobar birds. These results revealed that β-lactamases are circulating in the fowl population, including those living in remote locations with low anthropogenic activity. Furthermore, a partial clonal relationship of sequences of β-lactamases with human strains from the Indian subcontinent was observed, providing evidence of the inter-host transmission.

## Antimicrobial-resistant *Staphylococcus aureus*, enterococci, and *Campylobacter jejuni* of bovine origin

The European study conducted by Nemati et al. demonstrated the key clinical properties of bovine *Staphylococcus aureus*, including contagiousness and AMR. The bovine adhesion-like protein, encoded by *adlb* gene, was distributed among the different genotypes and clonal complexes (CCs) from ten European countries. Overall, *S. aureus* strains were inhibited by all antimicrobials tested, but some strains were resistant to important antimicrobials, including oxacillin. In this regard, MDR strains were detected in Belgium, Austria, Italy, and Germany. All penicillin-resistant strains showed the simultaneous presence of all *bla* operon genes searched. These results demonstrate that contagiousness and AMR seem to be correlated with different genotypes and CCs and that the prevalence of penicillin resistance is country dependent.

The review of Strong et al. addressed the factors associated with increase or decrease in the prevalence of antimicrobial-resistant enterococci applicable to the Canadian farm-to-fork beef continuum. For this proposal, articles discussing various factors associated with AMR were selected. The AMR was related to certain heavy metals and antimicrobial supplementation. In many instances, unique genes and phenotypic resistance patterns were nuanced. Furthermore, the interpretation of minimum inhibitory concentration and intrinsic resistance varied among enterococci. Several issues were identified, limiting the interpretability and comparison of factors at the broader One-Health scope. Therefore, studies focused on identifying research gap, as well as the standardization of laboratory methodologies are encouraged, which will contribute to future transdisciplinary projects and applications.

In *Campylobacter jejuni* strains, Goulart et al. investigated the growth kinetics and competition, as well as fluoroquinolone (FQ) resistance development. FQ-resistant strains had statistically significant increases over FQ-susceptible strains in growth in competition experiments carried out in mixed cultures without antimicrobials. In addition, FQ-susceptible strains developed resistance to ciprofloxacin more readily when exposed to low levels of the antimicrobial and at high initial bacterial cell density. Accordingly, these findings indicate that FQ-resistant strains may have a slightly higher fitness advantage over the FQ-susceptible strains and provides an explanation for the high prevalence of FQ-resistant *C. jejuni* strains in cattle production.

## The use of antimicrobials in livestock: trends and challenges

The Danish pig sector is one of the most important in the world and antimicrobial use (AMU) should be monitored. In this context, Moura et al. investigated which antimicrobials were used, how, and for which reasons. In 2020, there was practically no use of polymyxins, extended-spectrum cephalosporins, and fluoroquinolones. The use of orally administered antimicrobials for gastrointestinal indications in weaned piglets was highlighted. The substitution of group treatments for individual treatments, as well as the promotion of animal health and disease prevention, can enable further reductions in the AMU in the pig sector.

To understand the risk of AMR in livestock house aerosol and its association with AMU, Kobayashi et al. performed a study on the aerosol of the piggeries of Japanese farms. The results revealed that the AMR rate for critically important antimicrobials was positively associated with the AMU of them. The observed positive associations show that the AMR rate may be decreased by reducing the AMU. Therefore, these findings are expected to help establish countermeasures for AMR from aerosol bacteria in swine farms.

In Colombia, Roldan-Henao et al. evaluated the productivity and seroprevalence of *Salmonella* in pigs administered with organic acids (OA) compared to pigs given antimicrobial growth promoters (AGP). This pilot study indicated that administrating OA and cleaning the water pipes improve productivity in pigs and delay exposure to *Salmonella* species when compared with AGP. Although this study must be repeated before definite conclusions can be drawn, OA shows promise and may replace AGP, reducing AMU and AMR.

Indonesia is an important producer of broilers and empirical evidence has shown that the broiler industry uses excessive amounts of antimicrobials; however, quantitative data on AMU at the farm level is not available. Sani et al. compared on-farm AMU monitoring methods and assessed which monitoring method is most suitable for obtaining information on quantitative AMU at the farm level. Using four different indicators, this study demonstrated considerable differences in the ranking of AMU. Besides, collecting farm-level AMU data and adding it to a database can help with monitoring AMU trends.

In summary, these studies provided important findings on the transmission of AMR in Gram-negative and Gram-positive bacteria, as well as new insights into AMU in the veterinary sector. The Guest Editors of this Research Topic hope that these results will further motivate scientists to study and discuss the impact of livestock in the emergence, spread, and evolution of AMR.

## Author contributions

ES: Formal analysis, Writing—original draft, Writing—review and editing. WC-C: Formal analysis, Writing—original draft. KM: Formal analysis, Writing—original draft. JF: Formal analysis, Writing—original draft, Writing—review and editing.

